# 3D-Reconstructed Retinal Pigment Epithelial Cells Provide Insights into the Anatomy of the Outer Retina

**DOI:** 10.3390/ijms21218408

**Published:** 2020-11-09

**Authors:** Eloise Keeling, David S. Chatelet, Nicole Y. T. Tan, Farihah Khan, Rhys Richards, Thibana Thisainathan, Patricia Goggin, Anton Page, David A. Tumbarello, Andrew J. Lotery, J. Arjuna Ratnayaka

**Affiliations:** 1 Clinical and Experimental Sciences, Faculty of Medicine, University of Southampton, MP806, Tremona Road, Southampton SO16 6YD, UK; E.E.Keeling@soton.ac.uk (E.K.); nytt1e13@soton.ac.uk (N.Y.T.T.); fk3g15@soton.ac.uk (F.K.); rr5g15@soton.ac.uk (R.R.); tt1e16@soton.ac.uk (T.T.); A.J.Lotery@soton.ac.uk (A.J.L.); 2 Biomedical Imaging Unit, University of Southampton, MP12, Tremona Road, Southampton SO16 6YD, UK; D.S.Chatelet@soton.ac.uk (D.S.C.); P.Goggin@soton.ac.uk (P.G.); A.Page@soton.ac.uk (A.P.); 3 Biological Sciences, Faculty of Environmental and Life Sciences, Life Sciences Building 85, University of Southampton, Highfield Campus, Southampton SO17 1BJ, UK; D.A.Tumbarello@soton.ac.uk; 4 Eye Unit, University Hospital Southampton NHS Foundation Trust, Southampton SO16 6YD, UK

**Keywords:** retinal pigment epithelium (RPE), 3D reconstruction, retina, mouse, SBF-SEM, photoreceptors, imaging, age-related macular degeneration (AMD)

## Abstract

The retinal pigment epithelium (RPE) is located between the neuroretina and the choroid, and plays a critical role in vision. RPE cells internalise outer segments (OS) from overlying photoreceptors in the daily photoreceptor renewal. Changes to RPE structure are linked with age and retinopathy, which has been described in the past by conventional 2D electron microscopy. We used serial block face scanning electron microscopy (SBF-SEM) to reconstruct RPE cells from the central mouse retina. Three-dimensional-reconstructed OS revealed the RPE to support large numbers of photoreceptors (90–216 per RPE cell). Larger bi-nucleate RPE maintained more photoreceptors, although their cytoplasmic volume was comparable to smaller mono-nucleate RPE supporting fewer photoreceptors. Scrutiny of RPE microvilli and interdigitating OS revealed the angle and surface area of contact between RPE and photoreceptors. Bi-nucleate RPE contained more mitochondria compared to mono-nucleate RPE. Furthermore, bi-nucleate cells contained larger sub-RPE spaces, supporting a likely association with disease. Use of perfusion-fixed tissues ensured the highest possible standard of preservation, providing novel insights into the 3D RPE architecture and changes linked with retinopathy. This study serves as a benchmark for comparing retinal tissues from donor eyes with age-related macular degeneration (AMD) and other retinopathies.

## 1. Introduction

Retinal pigment epithelial (RPE) cells arise from the neuroepithelium, forming a monolayer between the neuroretina and the choroid. The RPE plays an important role in retinal homeostasis and acts as the outer blood retinal barrier. The monolayer consists of cells organised in a mosaic-like pattern (referred to as “cobblestone morphology”), reportedly numbering between 4.2–6.1 million cells in humans with a greater number in older eyes [1], and is supported by a thin porous tissue called the Bruch’s membrane (BrM) [2]. Analyses of the developing RPE layer in postnatal C57BL/6 mice showed 54,000 cells at day P15, which increased in size, in part through cell hypertrophy that continued beyond this period [3]. Structural specialisation of RPE cells has been described by conventional 2D electron microscopy (EM), which shows apical microvilli that wrap around outer segments (OS) of overlying photoreceptors. The internalisation and proteolytic degradation of OS by RPE cells is a key component of healthy vision, which becomes impaired with advancing age and the onset of retinal pathology [4]. The organisation of subcellular structures within RPE cells have also been described by 2D EM methods in the past. Melanosomes are localised apically, whilst mitochondria are observed predominantly in the basal third of RPE cells with the nucleus in proximity to the basal surface. The basolateral membrane is highly invaginated to increase the area for absorption and secretion, and also forms the innermost layer of the pentalaminar BrM [5,6]. Changes to RPE structure are associated with ageing and blinding diseases including a common cause of sight-loss termed age-related macular degeneration (AMD), as well as rare inherited retinopathies [2,7,8,9]. Elucidating the 3D anatomy of RPE cells could prove insightful in understanding the aetiology of sight-loss. We therefore took advantage of serial block face scanning electron microscopy (SBF-SEM), which, unlike conventional EM, can provide accurate qualitative and quantitative 3D information [10] to map the in situ ultrastructure of RPE cells.

## 2. Results

**Figure 1:**
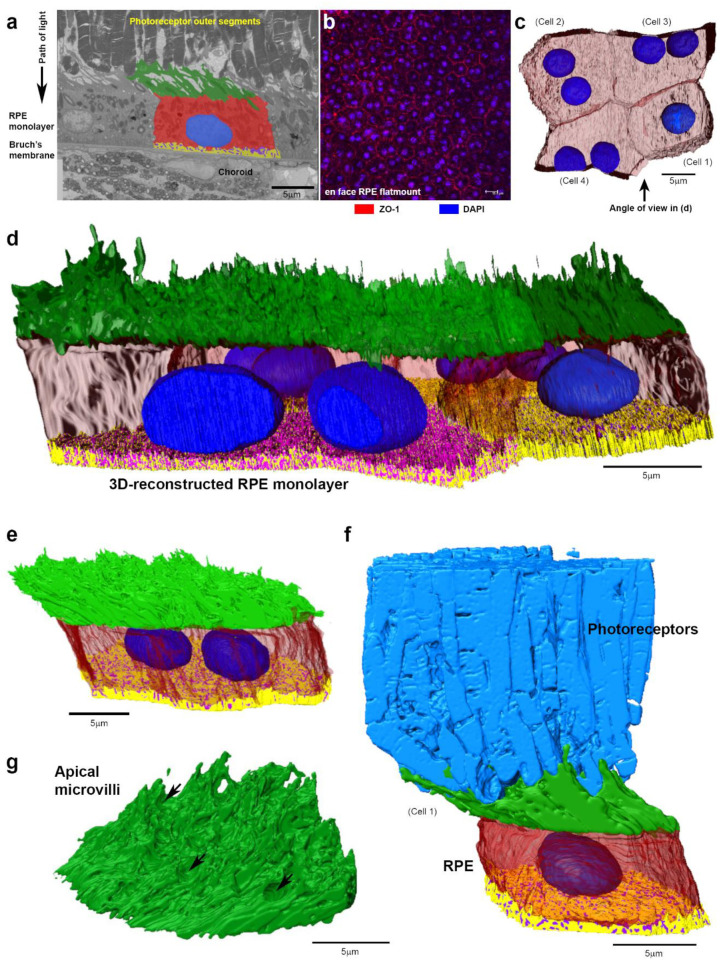
3D architecture of the retinal pigment epithelium (RPE). (**a**) A single image from a serial block face scanning electron microscopy (SBF-SEM) stack showing arrangement of tissues in the outer retina with a segmented RPE cell in relation to overlying photoreceptors and the underlying Bruch’s membrane and choroid. (**b**) Confocal microscope image of representative RPE flatmount from the central mouse retina showing cobblestone cell morphology and predominantly bi-nucleate RPE. Scale bar = 15 µm. (**c**) Top–down view of an RPE patch reconstructed in 3D. Individual cells are assigned numbers, which correspond to the cell in (**f**) and in Figure S1c. (data sourced from one of three SBF-SEM stacks obtained from eyes of 3 different animals). (**d**) 3D RPE monolayer (observed from the angle indicated in (**c**) showing apical microvilli (green), nuclei (blue) with transparent cytoplasm allowing visualisation of the convoluted basolateral membrane (yellow) with sub-RPE spaces (purple). (**e**) Side view showing rhomboid RPE cell with unidirectionally organised apical microvilli that interface with outer segments (OS) at the same angle. (**f**) 3D arrangement of photoreceptors in relation to the RPE. (**g**) Unidirectionally organised microvilli on the apical RPE surface and footprints (arrows) where photoreceptor OS terminate. Unless stated otherwise, scale bars = 5 µm.

Adult C57BL/6J mice were perfusion-fixed so that ocular tissues are preserved immediately upon death. Enucleated eyes were prepared for RPE flatmounts and SBF-SEM. Tissues were obtained from the adult mouse central retina, which was identified at a distance of approximately 400 μm dorsally from the centre of the optic nerve head [11,12], and 50 nm serial sections were reconstructed in 3D by manual and automatic segmentation (Figure 1a and supplementary AVI files).

Analysis of RPE flatmounts (Figure 1b) shows substantially more bi-nucleate cells in the central mouse retina compared to the periphery (Figure S1a,b). This difference becomes pronounced with age when comparisons are made between younger (3–6 months) and older animals (≥12 months). SBF-SEM stacks were obtained from three eyes of three different adult mice (3–6 months of age) and two of the stacks were segmented with the RPE and OS reconstructed in 3D. The third stack was used for visualisation purposes and for BrM thickness measurements. Although RPE cells are generally considered to adopt a hexagonal shape, 2D and 3D analyses show a mixed population of pentagonal as well as cuboidal shaped cells (Figure 1b,c). A 3D-reconstructed portion of the RPE monolayer shows a mixture of mono and bi-nucleate cells (Figure 1c,d). The cross-sectional view of RPE indicates a rhomboid rather than a columnar or rectangular morphology (Figure 1e). The 3D-reconstructed OS revealed details of interactions with individual RPE cells (Figure 1f, Table 1, and Figure S1c), where unidirectionally arranged microvilli interface with photoreceptors in the same direction to presumably maximise contact. Other studies have also used SBF-SEM to describe the organisation of apical RPE microvilli, which interdigitate with OS to maximise interactions [13]. The length and angle of RPE microvillus were 5.5 µm ± 1.1 SD and 143.0° ± 7.4° SD (*n* ˃ 100 measurements for each of the five cells from two eyes in different animals), respectively. Indentations on the microvilli bed showed footprints where OS terminate (Figure 1g). A manipulatable 3D RPE model is provided in supplementary Figure S2.

**Figure 2:**
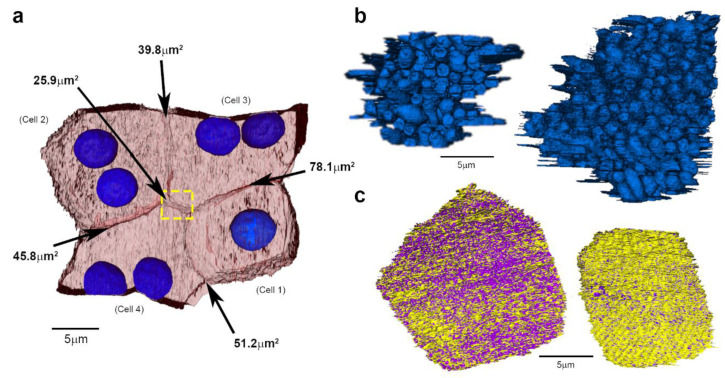
SBF-SEM of outer retinal morphology in the adult mouse. (**a**) Top–down view of 3D-reconstructed RPE monolayer indicating (arrows) contact area between neighbouring cells. The contact area at each interface is shown alongside. Individual cells are assigned numbers, which correspond to those in Figure 1f and in Figure S1c. (**b**) Bottom–up view showing the number of photoreceptors interacting with mono-nucleate (left) and bi-nucleate (right) RPE cells. (**c**) Top–down view of bi-nucleate (left) and mono-nucleate (right) cells showing infolds in the yellow basolateral membrane. Purple indicate sub-RPE spaces. Scale bars = 5 µm.

Analyses of contact between adjacent RPE cells revealed an average surface area of 48.1 µm^2^ ± 19.2 SD (*n* ˃ 352 measurements for interaction between each of the four cells in a single 3D-reconstructed RPE patch) (Figure 2a, Table 1). Three-dimensional data from fully reconstructed RPE cells show that the cytoplasmic surface area of bi-nucleate RPE were larger than their mono-nucleate counterpart (Table 1). Although smaller in size, the mono-nucleate RPE cell also supported 90 photoreceptors (Figure 2b, Table 1). The number of photoreceptors supported by each RPE cells ranged from 90–216 in the central mouse retina (Table 1). The height of RPE cells excluding microvilli was 6.7 µm ± 0.52 SD (*n* ˃ 100 measurements for each of the five cells from two eyes in different animals). After combining with the average microvilli length, the height of RPE in the central mouse retina was 12.2 µm ± 0.36 SD (*n* ˃ 100 measurements for each of the five cells from two eyes in different animals). Nuclei of mono and bi-nucleate RPE are ellipsoidal and of similar size (Table 1). When nuclei volumes were excluded, there was surprisingly no marked differences in the cytoplasmic volumes between mono and bi-nucleate RPE cells (mono-nucleate cell = 2079.2 µm^3^ vs. average for two bi-nucleate cells = 2231.5 µm^3^, considering whole cells only, Table 1). However, bi-nucleate RPE contained larger luminal spaces (basal infolds) underneath their basolateral membrane compared to mono-nucleate RPE (Figure 2c, Table 1).

**Figure 3:**
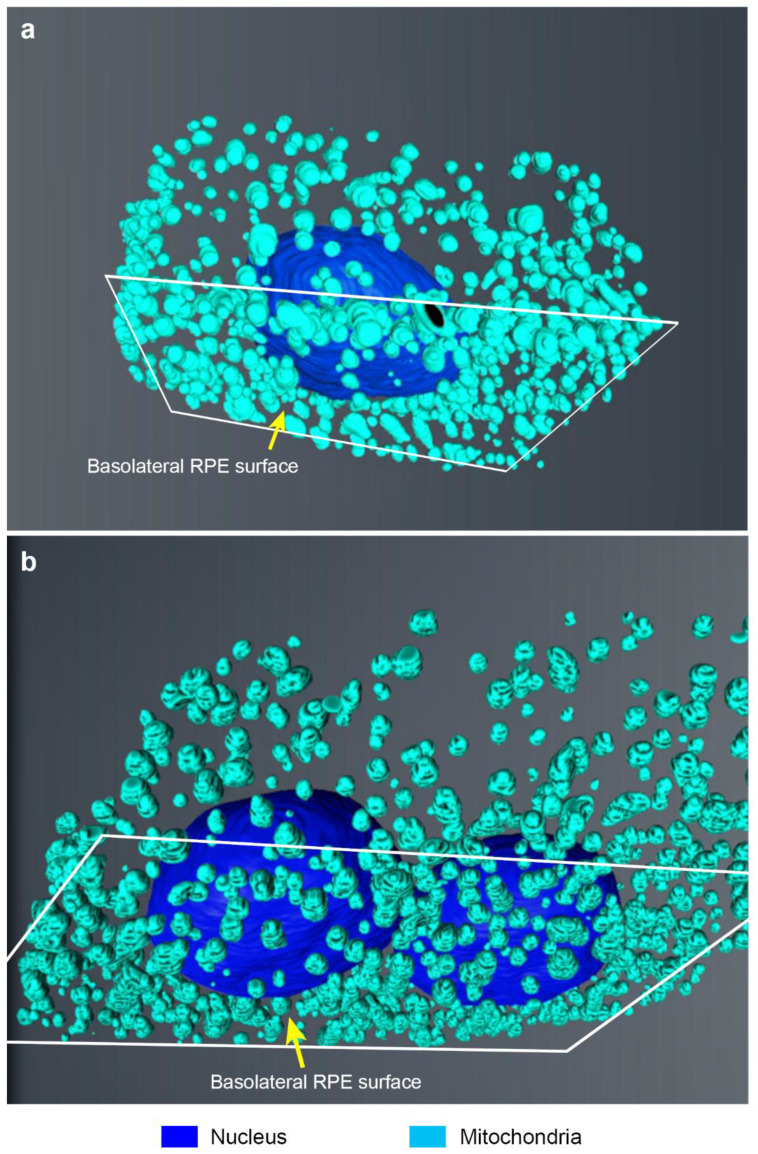
3D-reconstructed mitochondria in RPE cells. (**a**) View from below the basolateral membrane showing the distribution of mitochondria in mono-nucleate and (**b**) bi-nucleate RPE cells.

We also studied mitochondria in a subset of 3D-recontructed RPE cells. These organelles were observed predominantly in the basal region of cells (Figure 3 and Table 2). The mitochondrial volume in RPE was 11.5% as a proportion of the total cytoplasm. Furthermore, there was no correlation between mitochondria and the number of cell nuclei, other than a higher mitochondrial density in bi-nucleate RPE. Measurement of the underlying BrM thickness in SBF-SEM stacks was recorded as 524.4 nm ± 200.5 SD (*n* ˃ 100 measurements for each of the five cells from two eyes in different animals). Three-dimensional printing may be used to create a detailed model of an RPE cell (Figure S3).

## 3. Discussion

In this study, we exploited SBF-SEM to fully reconstruct RPE cells from the adult mouse retina for the first time. The use of SBF-SEM to reconstruct soft tissues in 3D was a laborious, time consuming process, which required delineating regions of interest (the cell body, nucleus, apical microvilli, etc.) in each micrograph throughout the sample stack. The scale of this task was reflected by the large number of micrographs analysed in this manner, which ranged between 1016–1838 individual images per stack. A caveat to this study is the limited number of samples that can be realistically analysed using this approach. However, results obtained using this technique have been shown to be accurate, highly reproducible, and insightful. For instance, similar approaches revealed initial counts and packing geometry of lipofuscin, melanolipofuscin and melanosomes in foveal RPE cells [14], impaired OS phagocytosis by RPE in enhanced S-cone syndrome patients [13], the arrangement of nascent rod OS disk membranes [15], the architecture of the basal RPE labyrinth [16] as well as novel organisational differences in the developing cornea [17], amongst other discoveries [15,18,19,20]. Where possible, we combined manual segmentation of RPE cells with partial automated segmentation of photoreceptor OS, which was possible due to their comparatively electron-dense nature. This resulted in OS being reconstructed in significantly less time compared to RPE cells.

Our study revealed novel insights into the 3D arrangement of RPE cells and associated tissues in the outer retina. Although the RPE adopts a hexagonal shape [21], 2D and 3D data show pentagonal as well as cuboidal shaped cells in the RPE monolayer of the mouse central retina. We also observed a large number of multinucleate RPE cells. Bi-nucleate cells constitute 2.1% of RPE in the developing mouse eye at P1, which increases to 26% by P30 [3]. Further studies showed that rodent RPE may have up to 85% bi-nucleate cells [22] with ~80% of RPE in the central retina reported to be of this type [23]. Multinucleation is thought to be triggered by atrophic cells in the RPE monolayer, whereby incomplete cytokinesis in surrounding cells could give rise to RPE with several nuclei [23]. As the death of RPE cells appear to be confined mainly to the macula, migration of RPE from the peripheral retina is also thought to occur as a compensatory mechanism [24]. Multinucleated RPE cells have been described in rodent models and in donor eye tissues [25,26,27,28]. For instance, the development of multinucleate RPE in a senescence-accelerated OXYS rodent model diminished following expression of the autophagy regulator P62/SQSTM1 [29]. Collectively, these findings indicate that multinucleated RPE cells are associated with various forms of retinopathy. Conventional EM studies in the rhesus monkey retina showed each RPE cell to support between 39–45 photoreceptors [30]. This number was often cited subsequently, until a more recent study using conventional EM approaches revealed that each RPE cell maintains ˃200 photoreceptors in the central mouse retina [12]. Our 3D studies also show that RPE cells support larger numbers of photoreceptors in the central mouse retina, with higher numbers of photoreceptors (~139) maintained by bi-nucleate RPE compared to 90 photoreceptors by mono-nucleate RPE. Studies using conventional methods in donor tissues describe how the cone density failed to show a consistent relationship with age or retinal location, and the total number of foveal cones remained stable [31]. The number of parafoveal rods show a decrease of 30% over adulthood [32]. The use of 3D approaches in future investigations will be informative in understanding the relationship between the number of photoreceptors per RPE cell as a function of age as well as location in mouse and human retinas, allowing for nuanced comparisons. The phenomenon of processing photoreceptor outer segments as part of the daily photoreceptor renewal is unique to RPE, whereby the distal 10% of OS are internalised every 24 h by RPE cells and degraded in the phagosome or autophagy-dependent lysosomal pathways [33]. The high photo-oxidative environment of the retina as well as defects in cargo processing have been shown to increase the proteolytic burden of RPE cells and contribute to retinopathy [4,34,35,36]. The age-related accumulation of lipofuscin within RPE, which fills ~20% of the cytoplasm by the eighth decade of life, places a further burden on cellular stress [37,38]. An interesting finding in our study was that cellular activities in bi-nucleate RPE appear to occur in the same cytoplasmic volume as mono-nucleate RPE, which support comparatively fewer photoreceptors. Multinucleate RPE cells may not only be susceptible to oxidative stress [23] but may also be proteolytically vulnerable. The likelihood of proteolytically stressed bi-nucleate RPE is supported by findings showing that there were no significant differences in the phagocytic activity between RPE cells with one or more nuclei [23]. Based on a limited sample number, our findings also indicate that bi-nucleate RPE cells maintain a larger number of photoreceptors compared to smaller mono-nucleate RPE but have the same cytoplasmic volume as the latter. Given the role of autophagy in the prevention of RPE multinucleation [29,39] as well as the importance of RPE proteostasis in the development of retinopathy [4,33], these results invite further studies that could reveal new insights into the aetiology of disease in the senescent retina.

The deposition of lipids and proteins underneath the RPE, termed drusen, is a key feature of early retinopathy [40]. The accumulation of debris in sub-RPE spaces as well as the presence of abnormal basolateral RPE infolds have been reported in long-term cultures of RPE cells, in mouse models of retinopathy and in donor AMD tissues [2,6,41,42]. Our 3D reconstructions show the presence of such sub-RPE spaces under the basolateral RPE surface, which were markedly increased in bi-nucleate RPE, indicating that these cells may be linked with pathology. Studies of mouse eyes using a similar SBF-SEM approach showed that basal infolds in RPE cells were organised into three distinct structural zones, which were largely devoid of organelles. Of these, the paracellular spaces between the basal infolds are closest to the cell body, and it opens out into cavernous cisternae. These zones are lost in a hierarchical manner with age and prematurely in a model of progressive retinal degeneration, including cisternal elements [16], supporting our observation that sub-RPE spaces under the basolateral RPE surface may be linked with pathology. Altered RPE basal infolds were also reported in an AMD-like mouse model, where autophagy-related *Atg5* or *Atg7* genes were inactivated specifically in these cells [39]. Similar observations were reported in RPE cells of 5xFAD transgenic mice, where high levels of retinal Amyloid beta (Aβ) recapitulate features of AMD [43]. Furthermore, equatorial drusen in donor tissues were reportedly associated with altered RPE cell size, morphology, as well as bi-nucleation [28]. Future studies using aged mice (≥12 months) or samples from rodent models of retinopathy could provide additional information such as comparisons with RPE size as well as morphology and insights into proteolytic stress and pathology. The cross-sectional view of RPE showed a rhomboid rather than a columnar or rectangular morphology, which could potentially maximise contact between adjacent cells. For the first time, we also obtained measurements of the surface area of cell–cell contact between adjacent RPE. Moreover, 3Dreconstructed images revealed nuanced details showing how each RPE cell interface with several adjacent RPE cells in the monolayer. The height of RPE cells in the central mouse retina was less than ~14 µm as reported in the human, although measurements in humans declined in eyes ˃70 years [44]. The resolution of the electron micrographs was sufficient to identify mitochondria, which we also analysed in a sub-set of RPE cells. Mitochondrial abnormalities in RPE are associated with ageing and retinopathy [45,46]. We observed mitochondria predominantly in the basal region of RPE cells, consistent with their reported distribution in mouse and primate RPE [45,47], and quantified the mitochondrial volume in RPE cells. Three-dimensional-reconstructed mitochondria show a heterogeneous population of different shapes and sizes, similar to those reported by conventional TEM in primate RPE cells [45] and as 3D images in mouse hippocampal neurons [48]. Another study using SBF-SEM describes how cisternal elements in the basal RPE labyrinth form membrane contacts with 3D-reconstructed mitochondria [16]. BrM thickness measurements in SBF-SEM stacks yielded data that were consistent with conventional EM measurements reported in the central mouse retina [12].

Although further work is needed to strengthen the findings of the current study, this first report exclusively on 3D-reconstructed RPE appears to indicate an association of bi-nucleate RPE cells with larger photoreceptor numbers and the potential to accumulate sub-RPE debris, which may help explain their reported association with drusen. Although mouse models are widely used to study retinal diseases [41,49], potential overlap with the aetiology and progression of human retinopathy must be considered with caution. Not only do rodents lack an anatomical macula equivalent to humans, but many models also fail to recapitulate focal pathology associated with maculopathies such as the geographic atrophic form of AMD or Sorsby fundus dystrophy [50]. Instead, their value lies as powerful in vivo tools to elucidate basic pathological mechanisms associated with sight-loss as well as screens for drug discovery. A caveat to SBF-SEM studies is the sample quality on which findings are based and presents particular challenges to studies of donor tissues with variable post-mortem times [51]. However, fixation of living mouse eyes provided the highest standard of tissue preservation, allowing unprecedented 3D views and information of RPE cells. These findings also provide a benchmark for comparing data from mice of different ages, mouse models of retinopathy, as well as studies of well-preserved healthy and diseased human donor tissues. Incorporating artificial intelligence software could also significantly reduce the time taken to reconstruct soft tissues in the future.

## 4. Materials and Methods

### 4.1. Animal Procedures

Adult C57BL/6J mice (males and females) were used in this study (*n* = 18). Animals were bred and maintained at the Biomedical Research Facility (BRF) at the University of Southampton, UK. Mice were maintained at 19–24 °C on a 12/12 h light/dark cycle and allowed access to standard laboratory chow and water ad libitum. Conventional cages containing Lignocel 2/2 (IPS Ltd., London, UK) bedding and environmental enrichment, housing no more than 10 animals per cage. Young mice were defined as animals between 3–6 months and old mice as ≥12 months of age. SBF-SEM studies were carried out using young mice. Animals were trans-cardially perfused with 0.9% saline prior to enucleation. Animal studies were overseen by the institutions’ Ethical Research Committee and carried out in accordance with the UK Animal (Scientific Procedures) Act of 1986. Experiments also conformed to the ARVO statement for the Use of Animals in Ophthalmic and Vision Research. The experimental protocol was approved by the University of Southampton Research Ethics Committee and work carried out under the UK Home Office project licence #P395C9E5F (licence approval date: 4 July 2016).

### 4.2. Preparation of Flatmounts

Mouse eyes were fixed in 4% PFA for 1 h prior to removing the anterior pole. Four incisions were made into the eyecup to allow the eye to lie flat. Flatmounts were placed on microscope slides (ThermoFisher, Loughborough, UK) and used for immunohistochemistry studies. The central mouse retina was identified at a distance of approximately 400 µm dorsally from the centre of the optic nerve head. Tissues were permeabilised in 0.1% Triton-X 100 for 30 min and subsequently blocked in PBS containing 1% BSA and 0.1% Tween for a further 30 min. Flatmounts were incubated with a ZO-1 primary antibody (Invitrogen, Inchinnan, UK, RRID: AB_2533456) diluted 1:100 in blocking buffer overnight at 4 °C. Cells were subsequently incubated for 1 h at room temperature with anti-rabbit Alexa Fluor 594 (Life Technologies, Inchinnan, UK, RRID: AB_142057) at 1:200. DAPI (1 µg/mL) was used to stain cell nuclei. Samples were mounted with a glass coverslip using Mowiol with Citifluor antifade. Images were acquired using a Leica SP8 confocal laser-scanning microscope (Leica Microsystems, Milton Keynes, UK). Z-stacks were taken for each field of view using sequential scanning and system-optimised settings. Image shown as maximal intensity projections and analysed using Fiji software [52] with statistical tests performed using GraphPad Prism (GraphPad, San Diego, CA, USA).

### 4.3. Preparation of Eyes for SBF-SEM

Mouse eyes were enucleated immediately following perfusion-fixation (0.9% saline with heparin) and placed in 3% glutaraldehyde in 0.1 M cacodylate buffer at pH 7.4 for 1 h. The eyes were dissected and the posterior pole washed in 0.1 M sodium cacodylate buffer (pH 7.4) plus 0.23 M sucrose and 2 mM calcium chloride for 10 min twice before being post-fixed in 1.5% potassium ferrocyanide in 0.15 M cacodylate buffer with 42 mM calcium chloride and 24% osmium tetroxide on ice for 1 h. Samples were rinsed in distilled water 5 × 3 min. Filtered thiocarbohydrazide solution (0.1 g in 10 mL distilled water, heated to 60 °C to dissolve) was added to the samples for 20 min at room temperature. Samples were rinsed in distilled water 5 × 3 min. Two percent osmium tetroxide was subsequently added for 30 min, after which the samples were rinsed again in distilled water 5 × 3 min, followed by uranyl acetate for 1 h. Finally, samples were placed in Walton’s lead aspartate solution for 30 min at 60 °C and rinsed in distilled water 5 × 3 min before being dehydrated in a graded series of ethanol (30%, 50%, 70%, 95%) for 20 min each, followed by absolute ethanol twice for 20 min. A link reagent acetonitrile was then applied for 20 min, after which samples were filtrated overnight in a 1:1 ratio of acetonitrile to Agar low viscosity (ALV). The following day, samples were submerged in fresh ALV resin for 6 h before being embedded and polymerised in ALV resin (Agar Scientific, Stansted, UK) at 60 °C for 16 h.

### 4.4. SBF-SEM

Resin blocks were loaded into a Reichert Ultracut E microtome (Leica Microsystems, Milton Keynes, UK). Blocks were trimmed to a trapezium around the sample using a razor blade. A glass knife was used to polish the surface and initial 90 nm thick sections cut and collected on 200 mesh carbon and formvar coated copper/palladium grids pre-treated with sodium hydroxide. The integrity and orientation of the sample as well as the quality of preservation was determined by conventional TEM, which was also used to identify a region of interest (ROI). A 500 µm^2^ block was cut from the original resin block and glued onto a roughened aluminium pin using silver loaded epoxy glue. This was left to polymerise overnight before being trimmed. Gold/palladium was sputter-coated on the block for 2 min. Glue was applied to the wide bottom edge of the block and loaded into the 3-View (Gatan, Abingdon, UK) with a Quanta 250 FEGSEM (ThermoFisher, Loughborough, UK). The diamond knife was bought into contact with the sample block and the electron chamber evacuated. The ROI was imaged at ×4512 with a scan resolution of 8192 × 8192, which equates to 4 nm per pixel. An accelerating voltage energy beam of 3.0 kV, spot size 3 or 3.5 and a vacuum of 40–50 Pa was used, and serial sections cut at 50 nm. Blocks from *n* = 3 eyes from three separate mice were imaged in this manner, with each stack containing 1016–1838 individual images. Single image acquisition time was 3 min and the total run times for collecting datasets were between 72–96 h.

### 4.5. Segmentation and 3D Reconstructions

The time consuming nature of SBF-SEM typically restricts the number of samples that can realistically be analysed. This particularly affects studies into soft tissues such as the RPE, which we manually segmented to preserve accuracy. Serial SBF-SEM stacks were aligned using a Fiji plugin “Register virtual stack slices” [53]. A Gaussian blur of 1.0 radius was applied to all images and the colour depth of 16-bit changed to 8-bit grayscale. Image size was reduced from 8192 × 8192 to 4096 × 4096 pixels. Entire RPE cells were visually identified and isolated into smaller image stacks, after which they were opened and segmented with the Fiji plugin TrakEM2. Colour-coded area lists corresponding to specific features in the images were assigned as follows: the RPE cell membrane (red), nucleus (blue), apical RPE microvilli (green), basolateral membrane (yellow) and sub-RPE spaces below the basolateral membrane (purple). Each area list of each image was then manually traced and filled-in. Once fully segmented, the volumetric and surface area information of each 3D object was calculated with TrakEM2. Fully segmented objects were then exported from TrackEM2, loaded into an Amira project (ThermoFisher, Loughborough, UK) and rendered in 3D. Mitochondria in RPE cells were identified based on the presence of a double membrane and cristae, and manually segmented in a separate TrackEM2 project. The segmented mitochondria were then exported into Amira and volumetric data extracted for analysis. Photoreceptor outer segments (OS) were more electron dense compared to the RPE monolayer, which enabled semi-automatic segmentation that considerably accelerated their 3D reconstruction. Semi-automatic segmentation was performed using the magic wand tool in AMIRA.

### 4.6. Analysis

Bruch’s membrane thickness, RPE microvillus length and the angle from the start of each RPE cell were measured in serial stacks at 50 slice intervals (*n* = 10 separate measurements per image). BrM measurements were recorded from under each of 5 RPE cells from two separate SBF-SEM stacks that were used to reconstruct the cells (*n* ˃ 100 measurements). A third SBF-SEM stack was also used, where BrM measurements (*n* ˃ 100 measurements) were recorded from under 3 RPE cells. A Fiji macro was written to measure the touching edge surface area of two adjacent volumes (two neighbouring RPE cells in the monolayer) and is shown below.

 object1 = “Untitled”; object2 = “Untitled”; Dialog.create(“Measuring shared area between”); Dialog.addString(“Object1:”, object1); Dialog.addString(“Object2:”, object2); Dialog.show(); object1 = Dialog.getString(); object2 = Dialog.getString();

 path = File.directory(); title_orig = getTitle(); getVoxelSize(width, height, depth, unit); pixelWidth = width; pixelDepth = depth; pixelUnit = unit;

 junctionArea_total = 0; junctionArea_array = newArray(0); imageNbr = nSlices();

 for(i = 1; i < imageNbr; i++){

   selectWindow(title_orig);   setSlice(i);

   run(“Duplicate...”, “title = Object1.tif”);   setAutoThreshold(“Default”);   //run(“Threshold...”);   setAutoThreshold(“Default dark”);   setThreshold(1, 1);   setOption(“BlackBackground”, false);   run(“Convert to Mask”);   rename(“Object1_binary.tif”);   run(“Dilate”);

   selectWindow(title_orig);   run(“Duplicate...”, “title = Object2.tif”);   setAutoThreshold(“Default”);   //run(“Threshold...”);   setAutoThreshold(“Default dark”);   setThreshold(2, 2);   setOption(“BlackBackground”, false);   run(“Convert to Mask”);   rename(“Object2_binary.tif”);   //run(“Dilate”);   //run(“Fill Holes”);

   imageCalculator(“AND create”, “Object1_binary.tif”,”Object2_binary.tif”);

   selectWindow(“Object1_binary.tif”);   close();   selectWindow(“Object2_binary.tif”);   close();

   selectWindow(“Result of Object1_binary.tif”);   setAutoThreshold(“Default dark”);   //run(“Threshold...”);   setAutoThreshold(“Default”);   run(“Create Selection”);

   getSelectionBounds(xRect0, yRect0, xWidth, yHeight);   selectionArray = newArray(xWidth*yHeight);   a = 0;   for(y = yRect0; y < yRect0 + yHeight; y++){      for(x = xRect0; x < xRect0 + xWidth; x++){         selectionArray[a++] = getPixel(x,y);      }   }

   selectionArray2 = newArray(0);   for(c = 0; c < selectionArray.length; c++){      if (selectionArray[c] > 254){         selectionArray2 = Array.concat(selectionArray2, selectionArray[c]);      }   }   nbrPixel = selectionArray2.length;   junctionLength = nbrPixel * pixelWidth;   junctionArea = junctionLength * pixelDepth;   junctionArea_array = Array.concat(junctionArea_array, junctionArea);   junctionArea_total = junctionArea_total + junctionArea;

   selectWindow(“Result of Object1_binary.tif”);   close();   }

 if (nResults > =0) {   run(“Clear Results”); } i = nResults;

 for (n = 0; n < junctionArea_array.length; n++){   setResult(“Slice”, i, n);   setResult(“Pixel_Unit”, 0, pixelUnit);

   setResult(“Surf.Area_stack”, 0, junctionArea_total);   setResult(“Surf.Area_slices”, i, junctionArea_array[n]);

   i = nResults; }   updateResults;

 saveAs(“Results”, path + “SharedSA_”+object1+”_”+object2+”.csv”); run(“Close”); selectWindow(title_orig); close(); exit(“ANALYSIS FINISHED”);

### 4.7. Statistical Analysis

Statistical tests were performed using GraphPad Prism (GraphPad, San Diego, CA, USA). Data were initially tested for normal distribution. Normally distributed data were analysed using a one-way ANOVA followed by Tukey’s multiple comparison test. Data are expressed as mean ± standard deviation (SD) with (*n*) representing the number of samples (indicated in figure legends). Statistical significance is denoted as * *p* ≤ 0.05, ** *p* ≤ 0.01, *** *p* ≤ 0.001 and *** *p* ≤ 0.0001.

## Figures and Tables

**Table 1:** Measurements from 3D-reconstructed data.

		Cell 1	Cell 2		Cell 3		Cell 4		Cell 5	
		Mono-Nucleate (Whole Cell)	Bi-Nucleate (Whole Cell)		Bi-Nucleate (Partial Cell)		Bi-Nucleate (Partial Cell)		Bi-Nucleate (Whole Cell)	
Cell cytoplasm	Volume (µm^3^)	2220.2	2360		1733		1803		2649	
	Surface area (µm^2^)	1794.9	2104		1572		1754.7		2055.6	
Microvilli	Volume (µm^3^)	528.5	877		60.9		181		1158	
	Surface area (µm^2^)	1556.4	2617.7		1814.5		3165.2		3146.3	
Nuclei	Volume (µm^3^)	141	146.8	144	139.5	126.5	128.2	138.1	123.6	131.6
	Surface area (µm^2^)	172.1	187.3	187	171.5	158.1	164.2	169.2	156.3	156.7
Basal infolds (sub-RPE spaces)	Volume (µm^3^)	40.9	164.1		92.4		116.7		172	
	Surface area (µm^2^)	537.9	1637.1		1682.2		1884.3		2505	
Surface of RPE microvilli in contact with photoreceptors (?m^2^)		51,462	90,000		48,732		73,118		242,797	
Number of photoreceptors supported per volume (?m^3^) of RPE cytoplasm		0.041	0.059		0.059		0.12		0.041	
Number of photoreceptors supported		90	132		102		216		108	

**Table 2:** Measurements of mitochondria from 3D-recontructed RPE cells.

	Mono-Nucleate RPE Cell	Bi-Nucleate RPE Cell
	(Cell 1)	(Cell 2)
Number of mitochondria	422	678
Average mitochondrial volume (nm^3^)	2.76 × 10^8^ ± 4.38 × 10^8^ SD	3.04 × 10^8^ ± 3.87 × 10^8^ SD
Volume of the smallest mitochondria (nm^3^)	2.35 × 10^4^	2.69 × 10^4^
Total mitochondrial volume (nm^3^)	1.17 × 10^11^	4.36 × 10^11^

## Supplementary Materials

Supplementary materials can be found at https://www.mdpi.com/1422-0067/21/21/8408/s1.
