# Supplementary material for: 3D-Reconstructed Retinal Pigment Epithelial Cells Provide Insights into the Anatomy of the Outer Retina
Source: Int J Mol Sci. 2020 Nov 9;21(21):8408. doi: 10.3390/ijms21218408 (PMC7672636; doi:10.3390/ijms21218408)
Supplement: Supplementary file 1 [file ijms-21-08408-s001.zip › ijms-979097-supplementary V2/ijms-979097-supplementary figures.pdf]

Supplementary information

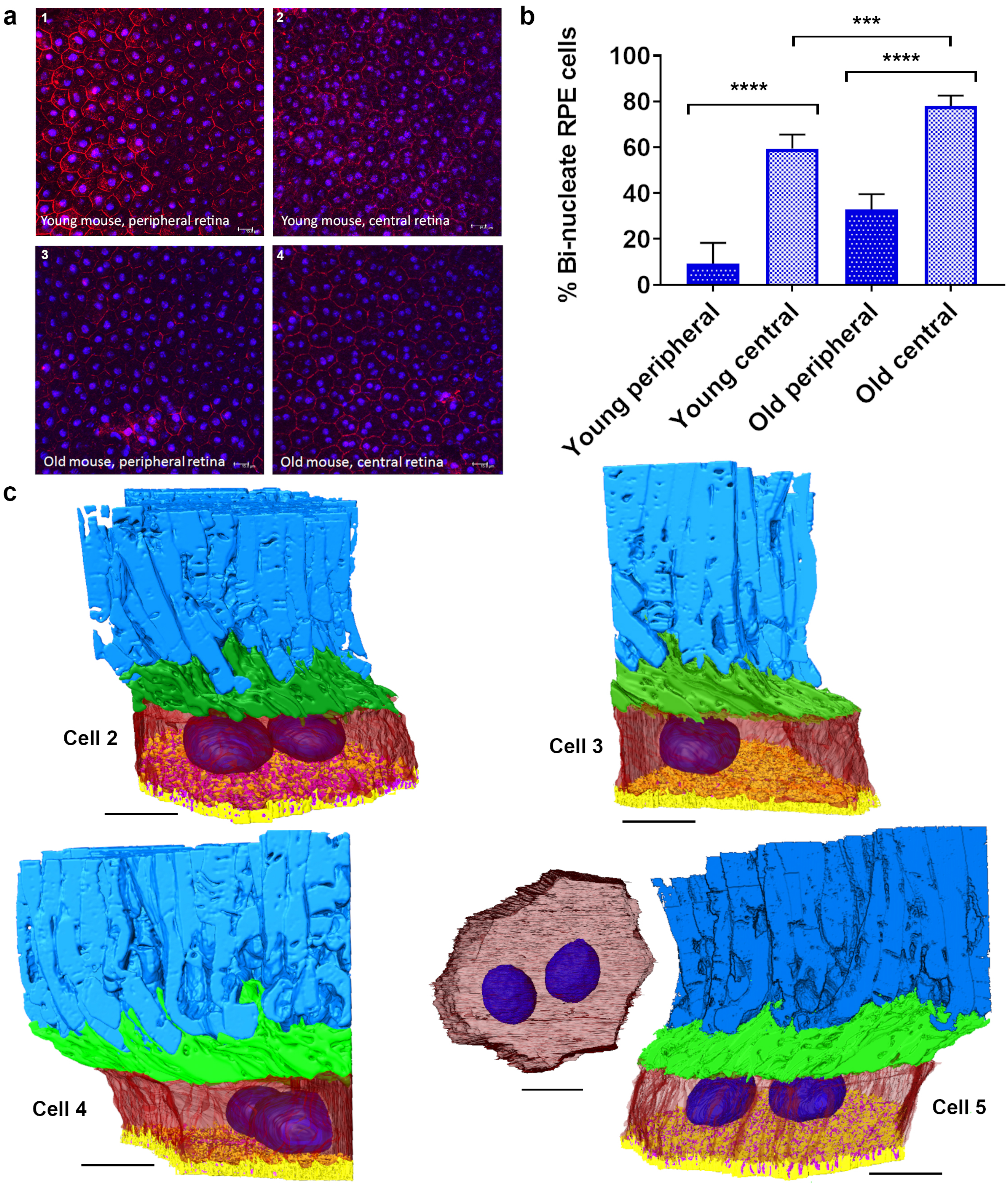

Supplementary Figure S1: 2D and 3D analysis of the retinal pigment epithelium (RPE). (a) Representative RPE flatmounts from the central and peripheral retinas of young (3 months) and old (12 months) mice show typical cobblestone morphology. ZO-1 junctional proteins are shown in red, whilst nuclei are stained with DAPI and appear blue. Scale bars correspond to 15μm. The number of bi-nucleate RPE appear to increase with age and between young vs. older eyes, particularly in the central retina. (b) This was confirmed by quantification of cell nuclei in blinded images. N=9/group from three different eyes of three separate animals. Significance was assessed using one-way ANOVA followed by Tukey's multiple comparison test where *** denotes $p \leq 0.001$ and **** indicates $p \leq 0.0001$. Error bars represent standard deviation (SD). (c) 3D-reconstructed photoreceptor outer segments and RPE cells showing their close relationship. Outer segments are shown in blue with the apical RPE microvilli in green. The RPE cytoplasm is shown in transparent red allowing visualisation of infolds (purple) below the basolateral membrane (yellow). Cells 1-4 are from a single patch of the RPE monolayer reconstructed in 3D and shown in Figure 1c. Cell 5 was segmented and reconstructed in 3D from the central retina of another animal. The top-down view of cell 5 is shown alongside its side view. Scale bars correspond to 500nm.

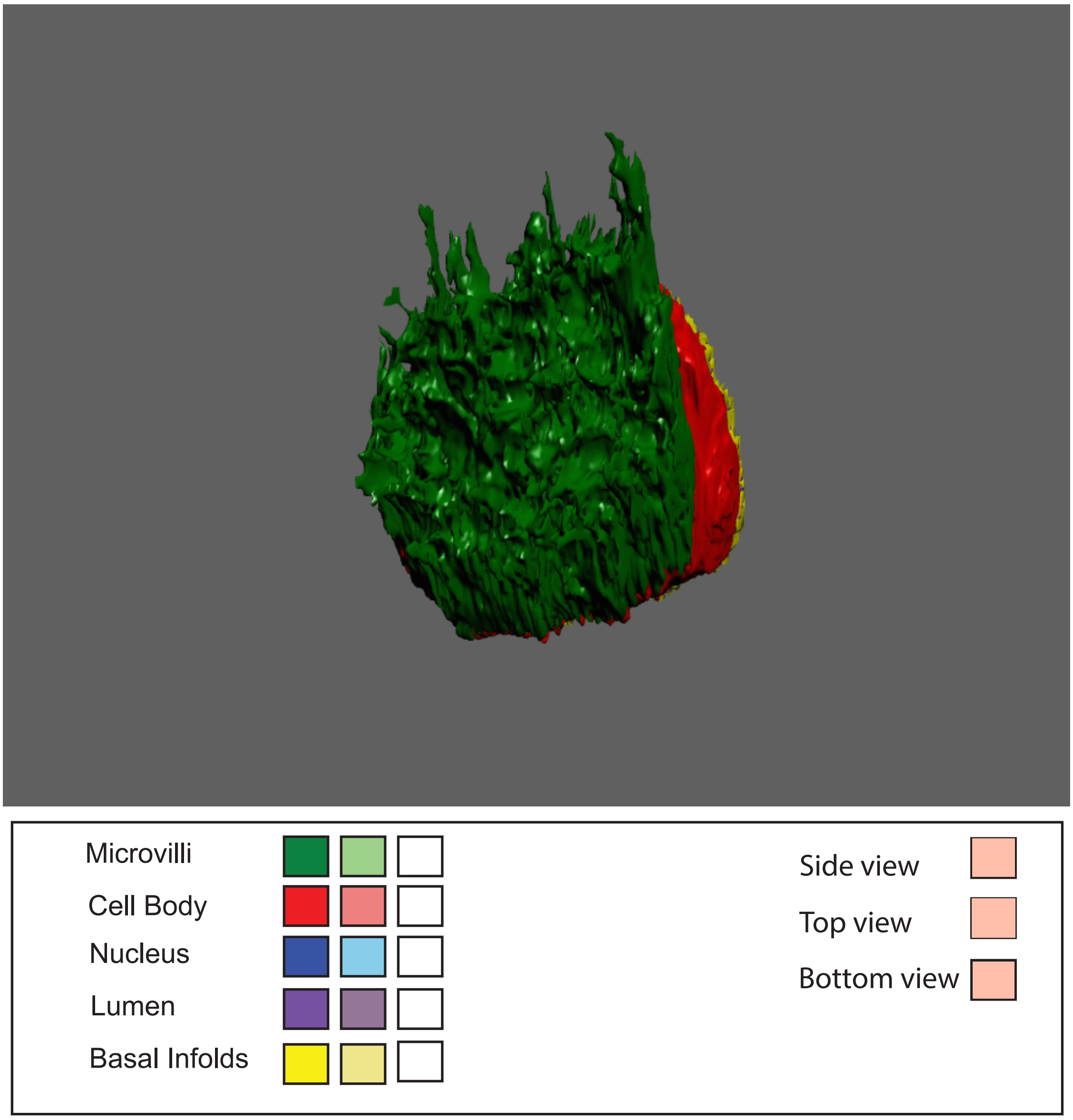

Supplementary Figure S2: Interactive 3D RPE cell. To activate the 3D interaction, click on the image followed by "Trust this document only once". The model can then be freely rotated and zoomed in (with the mouse wheel). Clicking on the different coloured buttons at the bottom will change the views and the transparency of the cell components.

To create the 3D PDF, meshes (surfaces) of each 3D objects were generated in Amira, exported as wavefront files (.obj) and imported into Meshlab (https://diglib.eg.org/handle/10.2312/LocalChapterEvents.ItalChap.ItalianChapConf2008.129-136). Each mesh was simplified, assigned a colour and exported as an obj file. All the obj files were opened into DAZ studio (DAZ 3D, USA), combined into one file and saved as a Universal 3d format (.u3d). A basic 3D PDF was created by inserting the 3D file into an Adobe Acrobat Pro document with activated 3D properties and adding Views and annotations. The more advanced 3D PDF with the buttons (as seen above) was created in Adobe Acrobat Pro by combining the basic PDF with a template made in Adobe Illustrator (layout of the coloured buttons, 3D object, legends, etc.). Functionality of the buttons was created using the Rich Media and Javascript tools in Adobe Acrobat Pro.

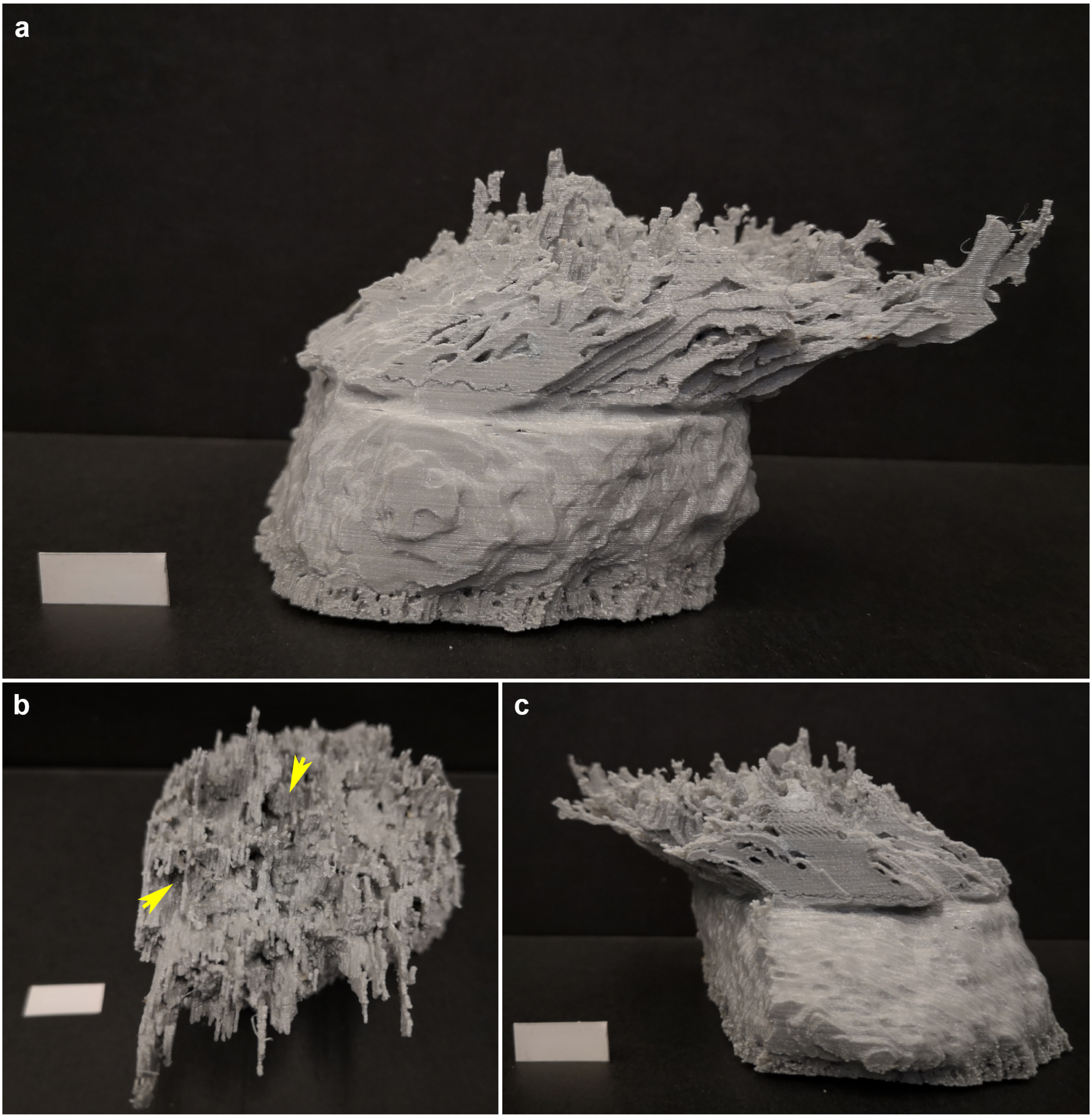

Supplementary Figure S3: Printing a 3D model of a reconstructed RPE cell. (a-c) The plastic model shown in the figure was created using an Ultimaker 3+ extended printer. Using the software Avizo, the cell body, microvilli, lumen and basal infolds were merged into one unique object and converted to a mesh. The mesh was exported as an stl file and inspected in Meshmixer 3.0 (AutoDesk, Inc.) for errors and manifold. If any errors were detected, the mesh was manually corrected and exported as an stl file. The inspected stl file was then imported in the 3D printer software Ultimaker Cura 4.0 where it was re-sized and oriented to fit the 3D printer dimensions. The 3D object was printed using polylactic acid (PLA) filament material. However, as the apical RPE microvilli formed fine structures and protruded at an angle, polyvinyl alcohol (PVA) filament was used as a supporting material, which dissolved in water once printing was completed after 48 hours. Note: photoreceptor OS footprints (arrows) in b, similar to those shown in Figure 1g. Scale bar corresponds to 2cm.
