# Supplementary material for: 3D-Reconstructed Retinal Pigment Epithelial Cells Provide Insights into the Anatomy of the Outer Retina
Source: Int J Mol Sci. 2020 Nov 9;21(21):8408. doi: 10.3390/ijms21218408 (PMC7672636; doi:10.3390/ijms21218408)
Supplement: Supplementary file 1 [file ijms-21-08408-s001.zip › ijms-979097-supplementary V2/Legend for Supplementary movies.pdf]

Supplementary movies

Supplementary Movie_1_Representitive SBF-SEM stack: Volumetric data through the mouse central retina showing structure of the outer retina in a single SBF-SEM stack.

Supplementary Movie_2_RPE segmentation: Representative SBF-SEM stack showing demarcation of RPE cell structure in different colours. Nuclei (blue), cell cytoplasm (red), apical microvilli (green), basolateral RPE membrane (yellow) and sub-RPE spaces (purple).

Supplementary Movie_3_3D printed RPE cell: 3D printing can be used to create a model of the RPE cell from SBF-SEM data. The scale of this model is shown by a rectangular prop, which corresponds to 2cm. Yellow arrows indicate footprints in the apical RPE microvilli where overlying photoreceptors terminate. Models of this kind can be scaled to any desired size and is an excellent tool for teaching purposes.
